# Preprocessing Strategies for Sparse Infrared Spectroscopy: A Case Study on Cartilage Diagnostics

**DOI:** 10.3390/molecules27030873

**Published:** 2022-01-27

**Authors:** Valeria Tafintseva, Tiril Aurora Lintvedt, Johanne Heitmann Solheim, Boris Zimmermann, Hafeez Ur Rehman, Vesa Virtanen, Rubina Shaikh, Ervin Nippolainen, Isaac Afara, Simo Saarakkala, Lassi Rieppo, Patrick Krebs, Polina Fomina, Boris Mizaikoff, Achim Kohler

**Affiliations:** 1 Faculty of Science and Technology, Norwegian University of Life Sciences, 1432 Ås, Norway; tiril.lintvedt@nofima.no (T.A.L.); johanne.heitmann.solheim@nmbu.no (J.H.S.); boris.zimmermann@nmbu.no (B.Z.); hafeez.ur.rehman@nmbu.no (H.U.R.); achim.kohler@nmbu.no (A.K.); 2 Norwegian Institute for Food Fisheries and Aquaculture Research (Nofima), 9291 Tromsø, Norway; 3 Research Unit of Medical Imaging, Physics and Technology, Faculty of Medicine, University of Oulu, 90220 Oulu, Finland; vesa.k.virtanen@oulu.fi (V.V.); Simo.saarakkala@oulu.fi (S.S.); lassi.rieppo@oulu.fi (L.R.); 4 Department of Applied Physics, University of Eastern Finland, 70211 Kuopio, Finland; rubina.shaikh@uef.fi (R.S.); ervin.nippolainen@uef.fi (E.N.); isaac.afara@uef.fi (I.A.); 5 Department of Orthopedics, Traumatology, Hand Surgery, Kuopio University Hospital, 70210 Kuopio, Finland; 6 Institute of Analytical and Bioanalytical Chemistry, Ulm University, 89081 Ulm, Germany; patrick.krebs@uni-ulm.de (P.K.); polina.fomina@uni-ulm.de (P.F.); boris.mizaikoff@uni-ulm.de (B.M.)

**Keywords:** preprocessing, sparse spectra, multiplicative signal correction, quantum cascade lasers

## Abstract

The aim of the study was to optimize preprocessing of sparse infrared spectral data. The sparse data were obtained by reducing broadband Fourier transform infrared attenuated total reflectance spectra of bovine and human cartilage, as well as of simulated spectral data, comprising several thousand spectral variables into datasets comprising only seven spectral variables. Different preprocessing approaches were compared, including simple baseline correction and normalization procedures, and model-based preprocessing, such as multiplicative signal correction (MSC). The optimal preprocessing was selected based on the quality of classification models established by partial least squares discriminant analysis for discriminating healthy and damaged cartilage samples. The best results for the sparse data were obtained by preprocessing using a baseline offset correction at 1800 cm^−1^, followed by peak normalization at 850 cm^−1^ and preprocessing by MSC.

## 1. Introduction

Infrared spectroscopy is an emerging technique in biomedical applications that has already demonstrated potential for diagnostics of various pathological conditions, such as cancer, osteoarthritis, and infectious diseases [1,2,3]. The method is easy to use, nondestructive and cheap which attracts even more attention to various applications in biomedicine [4,5,6,7]. This trend is further accelerated by the development of new photonic devices and light sources in the infrared, such as quantum cascade lasers (QCLs), that are either emitting light on a wide spectrum (frequency comb QCLs), tunable over spectral regions (wavelength-tuning QCLs) or have fixed wavelengths, and light-emitting diodes (LEDs) that cover narrow regions in the mid-infrared [8]. QCLs and LEDs with fixed wavelengths allow fast and relatively inexpensive measurements of samples. However, their use results in a loss of information, as only part of the broad mid-infrared region can be covered. For this reason, the collection of sparse wavenumber channels has become very common recently. QCL and optical parametric oscillator (OPO)-based imaging measurements can provide chemical images with high spatial resolution, reaching submicrometer resolution for photothermal infrared (IR) spectroscopy, such as in atomic force microscopy-infrared spectroscopy (AFM-IR). However, since the acquisition of infrared images with a full spectral depth is time-consuming, the measurements are usually limited to just a small number of wavelengths. Nevertheless, having a limited number of spectral variables is not necessarily detrimental in IR spectroscopy due to inherently high collinearity between variables. In fact, a number of studies have shown that sparse data is often sufficient for creating good discrimination models in IR spectroscopy [9,10,11,12,13].

As one might guess, sparse data have their challenges when it comes to analyses and modelling. One of the challenges in dealing with sparse data is preprocessing. Preprocessing involves removal of unwanted variation in the data that is due to white noise and other instrumental effects, as well as to detrimental interaction of infrared radiation and the sample. This type of detrimental interaction, such as scattering and multiple reflections of the infrared radiation, often hinders analysis of spectral data. Preprocessing of broadband spectral data is known to improve data modelling results, at least when classical machine learning methods are used [14,15,16,17]. Methods such as Deep Learning (DL) are known to perform well on the raw data without any preprocessing when the data set for training is big enough [18]. However, for most infrared datasets preprocessing is advantageous since they are of moderate or small size. Among the most used preprocessing approaches are baseline corrections, selection of the spectral region of interest, conversion of data into a derivative form, and normalization [19]. Model-based preprocessing strategies such as Extended Multiplicative Signal Correction (EMSC) and its variations are among the most widely used preprocessing methods for broadband spectroscopic data [16,20,21,22,23]. All the studies reporting preprocessing approaches for the spectral data are based on the preprocessing of the broadband spectra. To our knowledge there are no studies that describe approaches for spectral preprocessing of sparse data. The studies that report modelling using only few spectral variables are based on the sparse data obtained by selecting wavelengths of readily preprocessed broadband spectra.

In this study we compare different preprocessing methods for the sparse data obtained from the raw broadband Fourier Transform Infrared Attenuated Total Reflectance (FTIR-ATR) spectra. ATR-IR spectra are recorded by placing a sample in contact with internal reflection elements, such as diamond or zinc selenide crystals. In general, ATR-IR data is devoid of many unwanted spectral variations that are commonly encountered in transmittance IR data, such as scattering of the infrared radiation at a sample surface or interior (for example scattering artifacts in microparticulate samples), and multiple reflections of the infrared radiation within a thin sample (for example interference fringes in film samples) [24,25,26]. The datasets analyzed in the study are FTIR spectra of bovine and human cartilage, and the datasets were truncated into the sparse datasets by selecting seven wavenumbers. In this study, in addition to measured data, we suggest an approach to simulate broadband spectral data. The simulated data has the advantage of being more controlled and including specific information related to cartilage degeneration. In addition, by simulating the data, we can extend the amount of data significantly, which is an important aspect of the successful data analysis. The simulation method exploits variations in experimental broadband datasets and establishes a simulated dataset of healthy and damaged cartilage spectra. Cartilage broadband spectra were simulated using human data.

Optimal preprocessing of the spectral data was selected based on the performance of classification of samples into healthy and damaged cartilage groups. In addition to the binary classification into healthy and damaged samples, a multiclass classification model was established for the bovine data. Classification models were built using Partial Least Squares Discriminant Analysis (PLSDA) [27,28,29].

## 2. Results and Discussion

### 2.1. Histology Reference Data

Binary classification of cartilage samples of human, bovine and simulated data was done in this study. Cartilage damage was graded using the Osteoarthritis Research Society International (OARSI) grading system. Balance between groups is an important aspect of successful classification. When the classes are heavily imbalanced, linear models such as PLSDA tend to become biased towards the larger class. Therefore, to balance the groups of healthy and damaged samples, the cut-off $\theta_{OARSI}$ was selected to be equal to $\theta_{OARSI}=2$ for all datasets, where grade OARSI ≤ $\theta_{OARSI}$ belongs to healthy cartilage group, while grade OARSI > $\theta_{OARSI}$ belongs to damaged group. The thresholds were selected as close to early degeneration grade as possible while trying to keep the balance between groups. The groups of healthy and damaged samples for the bovine dataset were hardest to balance due to the number of damaged samples. This was due to the experimental set-up: the point of the experiment was to obtain strictly controlled cartilage samples damage, both mechanical and chemical. The OARSI grades were obtained for these samples thereafter. The treatment groups were, therefore, well balanced but not the OARSI grades because the amount of highly damaged samples was much bigger than the amount of the healthy samples. To achieve balance in the data, an oversampling technique was applied to the data. This helped to obtain better classification into healthy and damaged groups (see Figure S1 in Supplementary Materials). It is important to stress here that when building binary classification models into OARSI groups, only spectra of treatment samples were used (180 spectra in total). The samples of controls did not have OARSI grades and could not be used for the classification into healthy and damaged groups.

When multiclass classification into treatment groups G1–G5 and binary classification into treatment and control groups were done for the bovine data, oversampling was not needed because the treatment groups G1–G5 as well as treatment and control groups were balanced a priori. In Figure S2 the distribution of the samples for (a, b) human 1 and 2 datasets, and (c, d) bovine data before and after oversampling, are shown. The total number of spectra in healthy vs. damaged groups was 432 vs. 349 in human dataset 1, 433 vs. 358 in human dataset 2, 57 vs. 114 and 114 vs. 114 in the bovine dataset before and after oversampling. Therefore, the datasets used to establish binary classification models were quite well balanced. The distribution of the samples in the treatment groups to be used for the multiclass classification of the bovine data are presented in Figure S2e. These numbers are provided for the final datasets used for classification after a preclassification was applied to remove water and low-quality signal spectra.

### 2.2. FTIR Spectral Data

Prior to preprocessing, spectral quality was tested to remove spectra with too little cartilage signal and water spectra. Such spectra might be obtained during measurements when the contact between the cartilage samples and the ATR crystal is suboptimal or, in the case of excessive cartilage damage, when the spectra show signals of synovial and phosphate buffered saline (PBS) fluids surrounding the sample. In both cases, the obtained spectra are not useful for cartilage degeneration diagnostics, and such spectra can be considered as low-quality spectra and removed. Such preclassification resulted in nine spectra of the bovine dataset removed, which left 171 spectra in the treatment group and 180 in control for further analysis.

**Figure 1:**
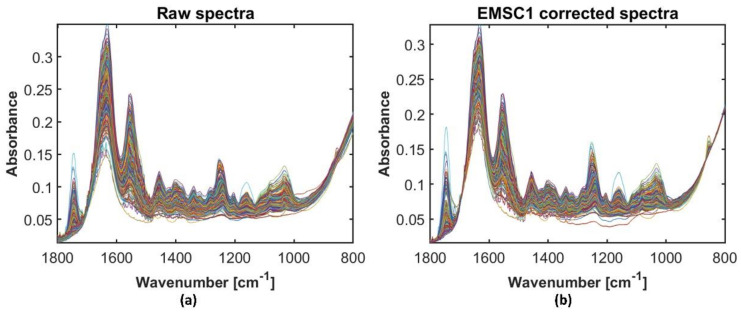
Human dataset 2 samples: (**a**) raw broadband spectra of after removing water spectra by EMSC pre-classification algorithm; (**b**) spectra preprocessed by the weighted EMSC1 (MSC plus linear term) model.

Some technical replicates of human data were removed due to technical issues. Then a preclassification method was applied to the datasets to remove water spectra. Twenty-one spectra of dataset 1 and six of dataset 2 were removed, which left 781 spectra in dataset 1 and 791 in dataset 2 for further analysis. The resulting spectra of dataset 2 after preclassification are presented in Figure 1a while dataset 1 spectra after the preclassification are presented in Figure S3.

**Figure 2:**
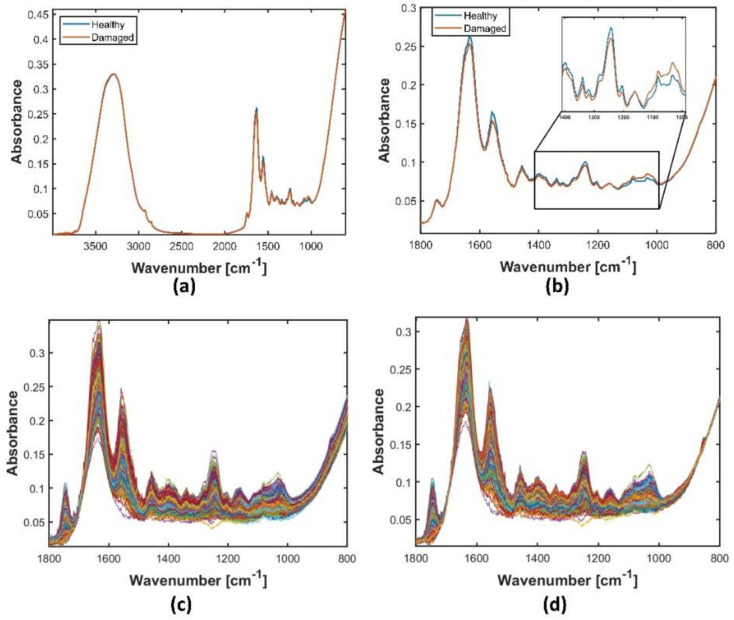
Simulated human cartilage spectra: mean spectra of simulated healthy (in blue) and damaged (in red) spectra showing (**a**) the full spectral range and (**b**) the fingerprint region; (**c**) all simulated apparent spectra in fingerprint region; (**d**) simulated spectra preprocessed by the weighted EMSC1 (MSC plus linear term) model.

In addition to the measured spectra of the cartilage samples, we simulated cartilage spectra. Spectral simulation was done by two independent PCA models for healthy and damaged group, respectively, where the threshold was set to $\theta_{\mathrm{OARSI}}=1.5$. Thus, healthy group samples had OARSI grades 0–1.5, while damaged group had OARSI grades > 1.5. The selection of the threshold is justified by the interest in detecting early cartilage degeneration. Simulated spectra are presented in Figure S4a,b for healthy and damaged groups, respectively. In total 1000 spectra were simulated: 500 for each group. The mean spectra of each of these groups as a full spectrum and in a fingerprint region as well as all simulated spectra are shown in Figure 2. We can observe that there are quite distinct differences between spectra of the groups. Some of these differences were observed in previously published studies [30]. This suggests that the simulation works well.

### 2.3. Preprocessing Strategies for the Broadband Spectra

To find optimal preprocessing of the broadband spectra, two types of measures of the preprocessing success were used: (i) visual inspection of the spectra, and (ii) classification results. The latter was based on the performance of binary models to classify healthy and damaged samples established using preprocessed broadband spectra and sparse spectra generated from the broadband spectra. Thus, eight datasets were used for that task: four sets of broadband spectra (two human sets, one bovine and one simulated dataset) and four sets of sparse spectra obtained from each set of the broadband spectra.

The seven different preprocessing options were compared: (1) simple preprocessing which includes baseline offset correction at 1800 cm^−1^, followed by peak normalization at 850 cm^−1^; (2, 3) MSC with and without weighting; (4, 5) EMSC1 with and without weighting; and (6, 7) EMSC2 with and without weighting. Broadband spectra preprocessed by EMSC1 with weights showed visually better results where all baseline variations were removed. The classification results for the weighted EMSC1 preprocessed broadband spectra were among the best results for all eight datasets (results not shown). Thus, the optimal preprocessing for the broadband spectra was identified to be weighted EMSC1 method. Figure 1b shows the spectra of the human dataset 2 preprocessed by weighted EMSC1 model. All broadband spectral datasets (human dataset 1 and 2, bovine and simulated data) preprocessed by this method were used as benchmark data for further analysis.

Sparse data were collected from the benchmark broadband data for each dataset. Different preprocessing strategies for the sparse raw data were then compared to the results of classification using sparse data of the benchmark.

### 2.4. Preprocessing Strategies for the Sparse Spectra

**Figure 3:**
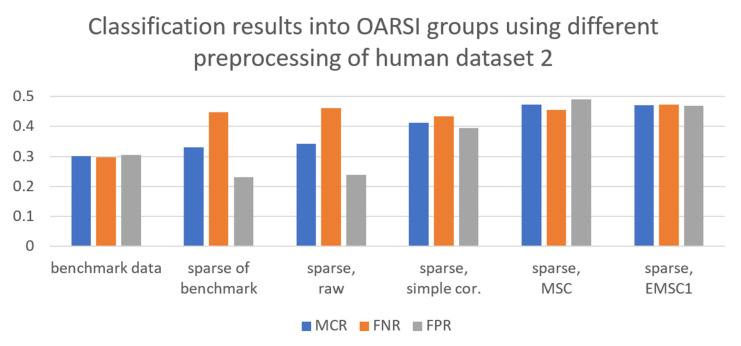
Binary PLSDA classification of healthy and damaged samples based on OARSI grades. Models were established using preprocessed spectra of human dataset 2. From left to right: (1) benchmark broadband data, (2) sparse spectra of the benchmark data, (3) sparse raw data, (4) sparse data with simple preprocessing, (5) sparse data preprocessed by MSC, (6) sparse data preprocessed by EMSC1. Overall misclassification rate (MCR = 1-Accuracy) as well as False Negative Rate (FNR = 1-Sensitivity) and False Positive Rate (FPR = 1-Specificity) for the damaged group are provided.

**Figure 4:**
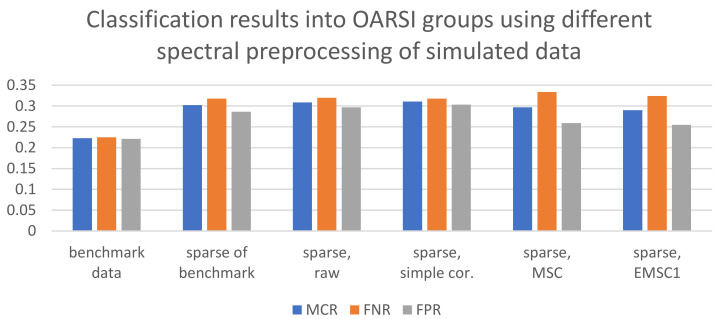
Binary PLSDA classification of healthy and damaged samples based on OARSI grades. Models were established using preprocessed simulated cartilage spectra. From left to right: (1) benchmark broadband data, (2) sparse spectra of the benchmark data, (3) sparse raw data, (4) sparse data with simple preprocessing, (5) sparse data preprocessed by MSC, (6) sparse data preprocessed by EMSC1. Overall misclassification rate (MCR = 1-Accuracy) as well as False Negative Rate (FNR = 1-Sensitivity) and False Positive Rate (FPR = 1-Specificity) for the damaged group are provided.

Different preprocessing strategies were applied to the sparse spectra obtained from the raw broadband spectra of human datasets 1 and 2, bovine dataset, and the simulated data. Binary classification models differentiating samples into healthy and damaged groups based on OARSI grades were built by PLSDA using preprocessed sparse data. The results are presented in Figure 3 and Figure 4 for the dataset 2 of human samples and for the simulated data, respectively, while Figures S5 and S6 of the Supplementary Materials present the results for the dataset 1 of human samples and bovine dataset, respectively. The misclassification rate (MCR = 1-Accuracy) provided in the figure shows the overall misclassification of the model, whereas false negative rate (FNR = 1-sensitivity) and false positive rate (FPR = 1-specificity) are provided for the class of damaged samples. MCR, FNR and FPR are selected to judge the model performances: better models have lower MCR, FNR and FPR. As can be seen from Figure 3, the best classification for human dataset 2 was obtained when the broadband spectra were used, with MCR, FNR and FPR close to 30%. The best preprocessing strategies for the sparse data were simple preprocessing (baseline offset at 1800 cm^−1^ with peak normalization at 850 cm^−1^) with MCR, FNR and FPR around 40%. Other approaches gave worse performances: MSC and EMSC1 gave almost 50% misclassification, while no preprocessing (raw data) gave very biased classification towards the healthy group (FNR raised to around 50% while FPR was close to 20%). Interestingly, sparse data of the benchmark spectra, i.e., spectra preprocessed before variables were selected, also gave quite unsatisfactory results with strong bias towards the healthy group.

The classification results for the human 1 dataset were quite similar irrespective of preprocessing strategy selected, with MCR around 30% (see Figure S5). When using sparse data, more samples were falsely classified as healthy: FNR was around 40% and higher, while FPR was between 20–25%. Among preprocessing strategies for the sparse data, the best approaches were the following: simple preprocessing, no preprocessing followed by MSC preprocessing. The biggest difference in classification results between broadband and sparse data was the balance of classification between groups of healthy and damaged samples in case of broadband spectra. Classification results for the sparse data tended to be biased towards healthy samples.

Results of classification into OARSI groups of healthy and damaged samples obtained for the bovine data are presented in Figure S6. MCR, FNR and FPR were around 30% for the broadband data but increased to 40% for the sparse data. The best preprocessing approach for the sparse data was simple preprocessing, followed by MSC and no preprocessing. These results were obtained when oversampling was used to balance the groups as it helped establish less biased classification models (see Figure S1). The oversampling made it possible to establish more balanced groups but did not add any additional variation in the data, as the spectra were simply duplicated. The results are presented for the Leave-OneCadaver-Out cross-validation where all replicates and duplicates corresponding to one cadaver were removed from a training set at each step of the cross-validation.

The multiclass classification results for the classification into the five bovine treatment groups are presented in Figure S7. Figure S7a presents a comparison of all preprocessing approaches across different treatment groups and overall misclassification. We can see that the best classified groups were G5 followed by G1 and G2. Worst classification was obtained for the G3 group which is the trypsin 30 min treatment. Figure S7b–g shows the same comparison across different preprocessing approaches applied to the spectra but separately for each of the metrics. The overall MCR is shown and the FNR for each group $G_{i},i=1,\ldots ,5$. We can see that the overall MCR was almost the same for all differently preprocessed sparse data and was around 40%. MSC performed only slightly better compared to other preprocessing approaches. The lowest MCR obtained was 20% for the benchmark data.

When looking at the groups G1–G5, we can see that MSC preprocessing in four cases (G1, G2, G3 and G5) produced lowest FNR among preprocessing approaches for the sparse data followed by simple correction. In cases of classes G2, G4 and G4, EMSC1 produced as good results as MSC preprocessing or even better results.

Binary classification into treatment and control groups for the bovine data is shown in Figure S8. Very good models were obtained with very low MCR, FNR and FPR around 10 to 20%. It is clear that MSC and simple correction were the best performing preprocessing approaches in this case with MCR, FNR and FPR around 17%, whereas the classification model on the raw data was worse. Interestingly, the model established on the sparse data of the benchmark spectra, where preprocessing is performed before variable selection, performed worse than preprocessed sparse data after selecting the variables.

Binary classification for the simulated data worked well both for the broadband data and sparse data with MCR, FNR and FPR around 22% and 30%, respectively (see Figure 4). The best preprocessing strategy was again simple preprocessing and raw sparse data. MSC and EMSC1 seemed to correct for some important variation related to sample quality. It is interesting that the sparse raw spectra and sparse spectra preprocessed by simple correction provided almost as good classification as the sparse data of the benchmark data.

To support the selection of the seven wavelengths as important for the classification of the cartilage diagnostics, regression coefficients of the PLSDA models are presented in Figure S9 in the Supplementary Materials. The models were obtained using benchmark data, i.e., preprocessed broadband spectra of human and bovine datasets by the weighted EMSC1 method. The regression plot confirms that the sparse data (respective wavenumbers are marked with red stars) had high coefficients in the PLSDA model. The exceptions were the outermost bands at 1800 cm^−1^ and 850 cm^−1^ which were selected for the preprocessing purposes, and therefore were not supposed to be important for the discrimination of the cartilage quality.

## 3. Methods

### 3.1. Measured Data

#### 3.1.1. Bovine Broadband Spectra

Bovine cartilage samples are commonly used as a model system for human cartilage samples [31]. The samples were acquired by an experiment in which cartilage damage was induced by a controlled mechanical and chemical treatment of bovine cartilage, as reported previously [32]. There were, in total, five different treatment groups, including three enzymatic damage groups: G1–collagenase 24 h treatment, G2–collagenase 90 min treatment, G3–trypsin 30 min treatment, and two mechanical damage groups: G4–surface abrasion and G5–impact loading. Each treatment was performed at assigned locations on the cartilage for the lateral and medial sections (for more detail see [32]). The dataset consists of measurements of 60 treated and 60 control samples, distributed across 10 bovine cadaver knees. For each treated sample, a separate control sample was extracted from an adjacent location. Phosphate-buffered saline (PBS) was put on top of the cartilage samples during measurements. The spectra were acquired using a custom-made ATR probe (Art Photonics GmbH, Berlin, Germany) that was connected to a Thermo Nicolet iS50 FTIR spectrometer (Thermo Nicolet Corporation, Waltham, MA, USA), equipped with a globar MIR source and a liquid nitrogen-cooled mercury cadmium telluride (MCT) detector [31]. The samples were measured in triplicates, with a new probe contact established for each measurement. Measurements were done with 2 cm^−1^ spectral resolution, digital spacing of 0.2411 cm^−1^, and averaging 64 scans over the range from 4000 cm^−1^ to 400 cm^−1^. The background spectrum of air was measured for each sample separately. Measurements were controlled by OMNIC software (Thermo Nicolet Corporation, Waltham, MA, USA). Thus, the dataset of treatment samples consists of 180 spectra in total with additional 180 spectra of controls.

#### 3.1.2. Human Broadband Spectra

Samples of nine human cadavers were obtained for this study [31]. During measurements, each sample was immersed in a PBS droplet. A PBS drop was put on top of a crystal and the inverted sample was submerged and measured. Spectra of human cartilage samples were recorded by two instruments and the resulting data were analyzed separately as dataset 1 and dataset 2. There were 269 samples in dataset 1 and 267 samples in dataset 2. The instrument used for the spectral acquisition of dataset 1 was a Bruker Alpha HR spectrometer and Bruker Alpha II HR spectrometer for the dataset 2 (Bruker Optics GmbH, Ettlingen, Germany), both equipped with a globar mid-IR source and a deuterated triglycine sulfate (DTGS) detector. The spectrometers were equipped with a Bruker Platinum ATR-sampling module (Bruker Optics GmbH, Ettlingen, Germany). For each spectrum, 128 scans were averaged. Spectra were recorded over the range from 4000 cm^−1^ to 600 cm^−1^ at a spectral resolution of 2 cm^−1^, and digital spacing of 1.0292 cm^−1^. All samples were measured in triplicates, resulting in 807 and 801 spectra in dataset 1 and dataset 2, respectively.

#### 3.1.3. Histology

After the experiments, the sample plugs were fixed in formalin, decalcified in ethylenediaminetetraacetic acid, and embedded in paraffin. Subsequently, 3-µm-thick histological sections were cut and stained using Safranin-O for qualitative evaluation of the cartilage damage.

One of the most used assessment systems for cartilage damage is the Osteoarthritis Research Society International (OARSI) histopathological grading [32]. It is a grading system based on tissue changes seen in stained histological sections, which reflect the extent of osteoarthritic progression of the tissue with 6 grades [33]. Other often used grading systems are the Mankin system [34] and that of the International Cartilage Regeneration & Joint Preservation Society (ICRS) [35]. In this study, all samples were graded by the OARSI grading system. All samples of each dataset were divided into groups based on their OARSI grades with the threshold $\theta_{\mathrm{OARSI}}=2$: healthy (OARSI grade 0–2) and damaged (OARSI grade > 2) to build classification models.

### 3.2. Simulated Broadband Spectra

In addition to experimental data, simulated broadband spectral data were also used in this study. The simulations were based on principal component analysis (PCA) using measured data of human samples of dataset 2. Full range spectra 4000–600 cm^−1^ were simulated with the digital spacing of 1.0292 cm^−1^.

To obtain the simulated data, we first corrected the measured spectral data by the extended multiplicative signal correction (EMSC) model with a linear term (referred in this paper as EMSC1, see Equation (3) and the method as described below. To capture potential differences in parameters of healthy (OARSI grade 0–2) and damaged samples (OARSI grade > 2), the EMSC1 parameters for the samples of these groups were stored separately $\beta_{i}=\left\{a_{i},b_{i},c_{i}\right\},\;i=1,2$. The means and standard deviations were calculated for each group separately, which formed the basis of two normal distributions of the EMSC1 parameters $N\left(\mu_{\beta_{i}},\;\sigma_{\beta_{i}}\right),\;i=1,2$, representing group specific physical effects in spectra. Furthermore, the EMSC1-corrected data were transformed such that the values were set to zero in the chemically inactive region 1780–2600 cm^−1^ by applying a window function based on Tukey [36] (see Figure S10). The EMSC1 corrected and transformed data were split into two groups $X_{i},\;i=1,2$: healthy and damaged samples, which were separately used for the simulation as follows:

1. PCA decomposition of matrix $X_{i}$ as $X_{i}=T_{i}P_{i}^{\prime }+E_{i}$ was done, where $T_{i}$ are scores and $P_{i}$ are loadings of matrix $X_{i}$.
2. Calculation of mean $\mu_{T_{i}}$ and standard deviation $\sigma_{T_{i}}$ of scores $T_{i}$ for the chosen number of loadings *A*.
3. New scores ${\tilde{T}}_{i}$ were drawn randomly from the respective normal distribution $N\left(\mu_{T_{i}},\sigma_{T_{i}}\right)$ calculated for each score $T_{i}$. The random drawing had a feedback loop which was activated if scores higher than the maximum or lower than the minimum obtained in experimental dataset were drawn. This was done to prevent very unrealistic score values being drawn.
4. The first set of simulated data were obtained by ${\tilde{X}}_{pure,i}={\tilde{T}}_{i}P_{i}^{\prime }$. These spectra generated for healthy and damaged groups separately were further merged into one dataset and corrected again by the EMSC1 method to avoid creating artificial physical effects by random recombination of loadings in the simulation.
  The resulting dataset ${\tilde{X}}_{pure}$ contained the final simulated pure absorbance spectra. To simulate apparent spectra which are “perturbed” by physical effects naturally present in the real data, the following was done.
5. Group specific EMSC1 variations were added to simulated pure spectra using parameters ${\tilde{\beta }}_{i}=\left\{{\tilde{c}}_{i},{\tilde{b}}_{i},{\tilde{d}}_{i}\right\}$ drawn from the distributions $N\left(\mu_{\beta_{i}},\;\sigma_{\beta_{i}}\right),\;i=1,2$.
6. The spectra were merged into one dataset and white noise vectors **w** were also added by randomly drawing from a uniform distribution $U\left(-\alpha ,\;\alpha \right)$ with the α level similar to experimental dataset.

Thus, the resulting simulated apparent absorbance spectra were obtained by


$${\tilde{X}}_{app,i}=f\left({\tilde{X}}_{pure,i},{\tilde{\beta }}_{i},w\right)\;\tilde{\beta }\sim N\left(\mu_{\beta_{i}},\;\sigma_{\beta_{i}}\right)\;w\sim U\left(-\alpha ,\alpha \right).$$

**Figure 5:**
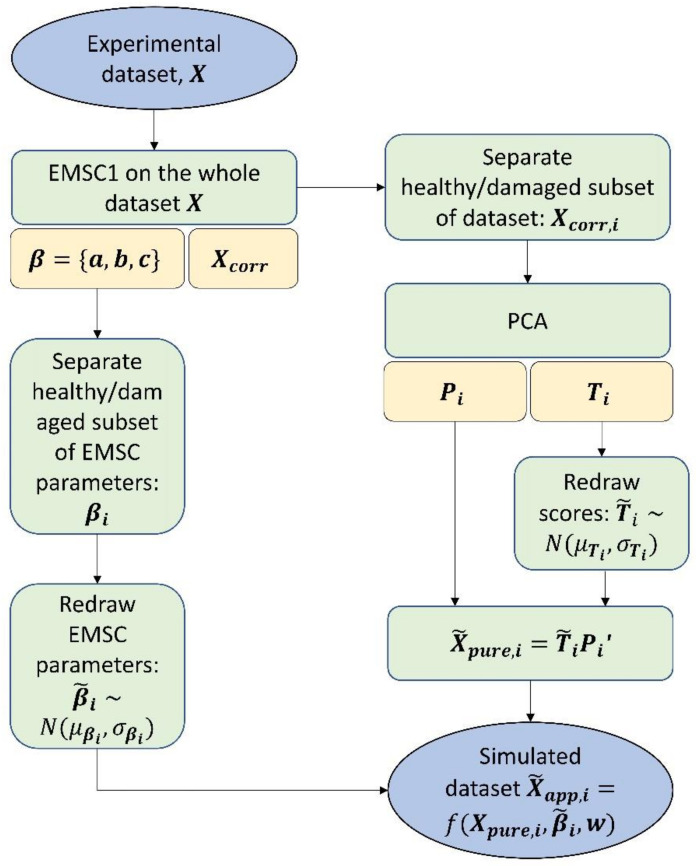
A flowchart for the PCA simulation of spectra. Blue blocks denote datasets, green blocks denote a computational action and yellow blocks denote results from the corresponding green block.

The schematic view of the algorithm is presented in Figure 5.

### 3.3. Sparse Spectra

**Figure 6:**
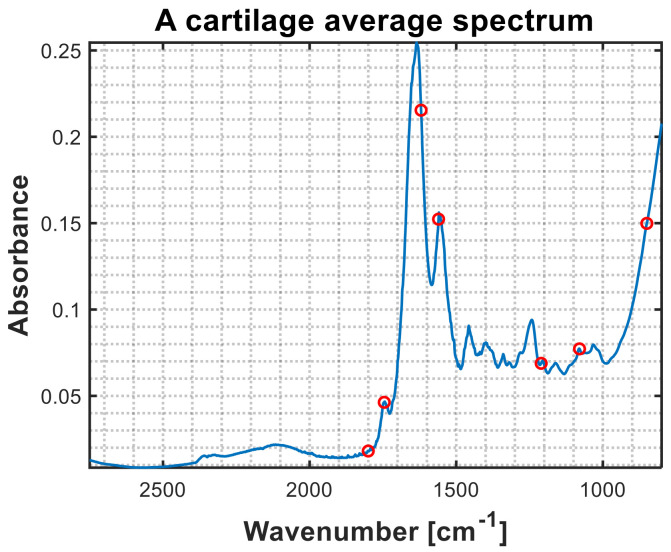
An average broadband spectrum of human cartilage obtained using dataset 2 in blue and the seven selected wavenumbers shown by red circles. The wavenumbers 1800, 1745, 1620, 1560, 1210, 1080, and 850 cm^−1^ were selected based on their relevance to cartilage quality assessment and spectral preprocessing.

The sparse data used in this study were obtained by selecting seven wavenumbers from broadband data, both measured and simulated. The seven wavenumbers selected were 1800, 1745, 1620, 1560, 1210, 1080, and 850 cm^−1^. These wavenumbers were selected as cartilage specific wavenumbers in the MIRACLE project for the production of custom-made fixed-wavelength QLC lasers. The positions of the preselected wavenumbers are shown on the broadband spectrum of human cartilage in Figure 6. The choice of the wavenumbers was based on the relevance of the wavebands for the discrimination of the cartilage quality, as well as the usefulness of the wavenumbers for the preprocessing of the spectra prior to the modelling.

The first wavenumber at 1800 cm^−1^ was selected as a reference variable for the baseline absorbance. As can be seen from the Figure 6, the spectral region between 2750 and 1780 cm^−1^ is mostly devoid of strong chemical absorbance by the samples, and thus it can be used for determining baseline absorbance. However, strong absorbance between 2500 and 1900 cm^−1^, caused by the ATR diamond crystal, results in a relatively low signal-to-noise ratio in this region, and therefore 1800 cm^−1^ was selected as an optimal baseline reference variable. Five out of seven wavenumbers were selected based on cartilage-specific absorbance, namely: 1745 cm^−1^, corresponding to the C=O stretching vibration of lipids present in the cartilage and synovial fluid; 1620 cm^−1^, corresponding to the C=O stretching vibration (amide I) of collagen; 1560 cm^−1^, corresponding to the C-N-H stretching and bending vibration (amide II) of collagen; 1210 cm^−1^, corresponding to the O=C-N-H stretching and bending vibration (amide III) of collagen, and 1080 cm^−1^ corresponding to the C-O stretching vibration of carbohydrate residues in collagen and proteoglycans [37,38]. The seventh wavenumber, at 850 cm^−1^, is a band related to librations of water in the cartilage and synovial fluid. This wavenumber was selected as a reference variable since it is assumed that the chemical absorbance of water is mainly invariant in all the samples, and therefore it could be used for normalization of all measured variables.

### 3.4. Spectral Preprocessing and Preclassification Strategies

A preprocessing approach that is widely used in infrared spectroscopy in Extended Multiplicative Signal Correction (EMSC) and Multiplicative Signal Correction (MSC). The MSC correction model is given by

(1) $$Z\left(\tilde{\nu }\right)=a+b\cdot Z_{ref}\left(\tilde{\nu }\right)+\varepsilon \left(\tilde{\nu }\right)$$

where the parameter a is the baseline shift, b is the optical path length. The model spectrum $Z_{ref}\left(\tilde{\nu }\right)$ represents a reference spectrum, e.g., a mean spectrum, and $\varepsilon \left(\tilde{\nu }\right)$ represents the unmodelled residual [20,21,39]. An Extended MSC (EMSC) model is obtained when in addition to baseline and multiplicative parameters a and b, wavelength-dependent terms, such as linear and quadratic terms c and d, are included or when chemical spectra are added to the EMSC model [16,38]. EMSC in its basic form writes as

(2) $$Z\left(\tilde{\nu }\right)=a+b\cdot Z_{ref}\left(\tilde{\nu }\right)+c\tilde{\nu }+d{\tilde{\nu }}^{2}+\varepsilon \left(\tilde{\nu }\right)$$

We used the following convention to refer to EMSC models with different orders of polynomials. EMSC1 is the model obtained when MSC is extended by linear term $c\tilde{\nu }$ only

(3) $$Z\left(\tilde{\nu }\right)=a+b\cdot Z_{ref}\left(\tilde{\nu }\right)+c\tilde{\nu }+\varepsilon \left(\tilde{\nu }\right)$$

while EMSC2 is the version according to Equation (2). Following this convention EMSC0 is identical to MSC (Equation (1)).

In a weighted EMSC we applied weighting of spectral regions when estimating the EMSC parameters. This strategy was followed when we needed to up-weight or down-weight some of the regions in the spectra when estimating the EMSC parameters. In this paper, we up-weighted regions 1800–1780 cm^−1^ and 900–800 cm^−1^. The weights are presented in Figure S11. This weighting strategy was chosen to correct baseline variations.

The following preprocessing strategies for the broadband spectra were tested: (1) simple preprocessing which includes baseline offset correction at 1800 cm^−1^, followed by peak normalization at 850 cm^−1^; (2, 3) MSC with and without weighting; (4, 5) EMSC1 with and without weighting, and (6, 7) EMSC2 with and without weighting. The broadband data preprocessed by weighted EMSC1 are referred to as the benchmark data in this study.

Different preprocessing strategies were evaluated for the sparse data in this study. The sparse data were collected from raw broadband spectra of human dataset 1 and 2, the bovine dataset and the simulated dataset. The sparse raw data were preprocessed by three approaches: (1) simple preprocessing which includes baseline offset correction at 1800 cm^−1^, followed by peak normalization at 850 cm^−1^; (2) MSC, and (3) EMSC1. The EMSC2 approach was not used in this study for two reasons. First, EMSC with its four parameters to be estimated for the sparse spectra containing seven channels has too few degrees of freedom to be implemented. Second, the spectra in this study were ATR-FTIR spectra and are thus devoid of complex spectral variations caused by radiation-sample interactions (such as scattering artifacts and interference fringes), that would have required correction by nonlinear EMSC terms. Simple baseline effects that can occur in ATR spectra can be corrected by MSC or EMSC1. To compare the performance of the preprocessing approaches for the sparse data, classification models were built, and the results were compared to the classification results using sparse data of the benchmark data - broadband spectra preprocessed by EMSC1 with weights.

The MSC method was also used in this study for preclassification of spectral data. The preclassification method was proposed in [40] and is based on the MSC model where a particular reference spectrum of interest $Z_{ref}$ can be used to preselect spectra of similar chemical fingerprint. In our case, a water spectrum was used as a reference spectrum $Z_{ref}$ in the MSC model to capture low signal cartilage spectra and water spectra. In the preclassification, the residual of the MSC model was used to decide whether the spectrum was a water spectrum or not based on a predefined threshold. For more details on the method see [40].

### 3.5. Classification Modelling

To establish classification models, the partial least squares discriminant analysis (PLSDA) method was used. In PLSDA, a classifier is established by regressing the matrix of indicator variables (dummy matrix representing each sample class belongingness) Y onto the matrix of FTIR spectra X [27,29,41]. The underlying idea of the method is to find new latent PLS variables for X and Y for which the covariance matrix of X and Y is maximized [42]. The new variables are then used to establish a PLSDA model, for which the optimal number of PLS components in X and Y needs to be found. This optimization process is usually done by cross-validation. In this study, Leave-OneCadaver-Out cross-validation was used. The optimal number of PLS components (*A_Opt_*) corresponded to the maximum accuracy of the group with the lowest classification accuracy. Three different models were used in this study. First, binary classification using OARSI groups of the cartilage samples for human, bovine and simulated data. Second, multiclass classification into treatment groups of bovine samples: five groups G1–G5. Third, binary classification into treatment and control groups for bovine samples.

Thus, in the case of binary classification *A_Opt_* was obtained by $\underset{A_{Opt}}{\max}\left(\min\left(Accuracy\left(healthy\right),\;Accuracy\left(damaged\right)\right)\right)$, while in the case of multiclass classification by $\underset{A_{Opt}}{\max}\left(min\left(Accuracy\left(G_{i}\right)\right)\right),i=1,\ldots ,5$.

Due to imbalance in the OARSI groups of bovine data, an oversampling technique was used [43]. Oversampling is a well-known technique in machine learning and is used to deal with highly imbalanced data. For this data the subset of healthy samples was underrepresented. To help establish binary classification by PLSDA, the data of healthy samples was simply duplicated which did not introduce any new variation into the dataset but increased the number of samples to balance the groups. Leave-OneCadaver-Out cross-validation ensured that all replicates and duplicates corresponding to one cadaver were removed from a training set at each step of the cross-validation.

## 4. Conclusions

The study was done in connection with the Horizon 2020 Research and Innovation Programme (H2020-ICT-2016-2017) project MIRACLE, in which the primary goal was to build a system based on seven QCL lasers in mid-IR region and an ATR probe to assess cartilage quality. In this study we evaluated different preprocessing strategies for such sparse data using spectral data of FTIR-ATR.

The study shows that when an optimal preprocessing of the spectra is selected, the classification success using sparse data does not drop strongly compared to models where the broadband spectral data were used. The best preprocessing for the sparse data in this study seemed to be simple baseline correction by a baseline offset at 1800 cm^−1^ and peak normalization by the water band at 850 cm^−1^. In some cases, preprocessing of sparse data by MSC showed good results as well. However, in most cases when extended by more terms, as in EMSC1 by a linear term, the performance of the classification dropped. Since the EMSC1 estimates three parameters from the seven selected wavenumbers in the sparse data, relevant chemical or physical information related to cartilage quality, may be modeled and removed from the data.

The data used in this paper were obtained from FTIR-ATR measurements, which are highly reproducible, and mostly devoid of undesirable spectral variations caused by radiation-sample interactions, such as scattering artifacts and interference fringes. Therefore, advanced spectral preprocessing approaches are normally not needed in FTIR-ATR spectroscopy. Since the MIRACLE fiber-optic probe will be based on a similar ATR measurement setup, it is expected that the sparse data will have a low level of undesirable spectral variations. When data are obtained by other techniques, such as transmission, transflection and specular reflection measurements, EMSC preprocessing may be of advantage. However, more of the spectral variables would be needed to apply an EMSC model with four parameters to have enough degrees of freedom for further analysis.

The spectral simulation algorithm proposed in the study was shown to create cartilage spectra with distinct patterns between healthy and damaged cartilage. The classification of these spectra worked very well both for the broadband spectra and the sparse data.

The results of the study were obtained using cartilage spectra; however, they can be generalized and applicable to different type of samples, and even other types of spectral data. In the era of rapid development of cheap photonic solutions, the preprocessing approaches for sparse spectral data will show their potential.

## Supplementary Materials

The following supporting information can be downloaded online. Figure S1: Classification results for the bovine data when no oversampling was applied (a) and for oversampled data (b); Figure S2: Distribution of samples in OARSI groups: healthy vs. damaged of human 1 (a), human 2 (b), and bovine data before oversampling (c) and after oversampling (d), distribution of the samples in the treatment group for the bovine data in (e); Figure S3: Raw broadband spectra of human dataset 1 samples after removing water spectra by spectral preclassification method based on EMSC; Figure S4: Simulated apparent spectra of human (a) healthy and (b) damaged cartilage; Figure S5: Classification results into OARSI groups using different preprocessing of human dataset 1; Figure S6: Classification results into OARSI groups using different preprocessing of bovine dataset; Figure S7: Classification into treatment groups using different preprocessing of bovine dataset; Figure S8: Classification results into treatment and control groups using different preprocessing of bovine dataset; Figure S9: Regression coefficients of the PLSDA models obtained on the benchmark broadband spectra of human dataset 2 (a) and bovine dataset (b); Figure S10: Window function Tukey used in spectral simulation; Figure S11: Wavenumber-dependent weights used for preprocessing of broadband spectra by EMSC1.
