# Supplementary material for: Preprocessing Strategies for Sparse Infrared Spectroscopy: A Case Study on Cartilage Diagnostics
Source: Molecules. 2022 Jan 27;27(3):873. doi: 10.3390/molecules27030873 (PMC8839829; doi:10.3390/molecules27030873)
Supplement: Supplementary file 1 [file molecules-27-00873-s001.zip › molecules-1552603-supplementary.pdf]

Supplementary materials

Preprocessing strategies for sparse infrared spectroscopy: a case study on cartilage diagnostics

Valeria Tafintseva [1,*], Tiril Aurora Lintvedt [1,2], Johanne Heitmann Solheim [1], Boris Zimmermann [1], Hafeez Ur Rehman [1], Vesa Virtanen [3], Rubina Shaikh [4,5], Ervin Nippolainen [4], Isaac Afara [4], Simo Saarakkala [3], Lassi Rieppo [3], Patrick Krebs [6], Polina Fomina [6], Boris Mizaikoff [6] and Achim Kohler [1]

[1] Faculty of Science and Technology, Norwegian University of Life Sciences, Ås 1432, Norway
[2] Norwegian Institute for Food Fisheries and Aquaculture Research (Nofima), Tromsø 9291, Norway
[3] Research Unit of Medical Imaging, Physics and Technology, Faculty of Medicine, University of Oulu, Oulu 90220, Finland
[4] Department of Applied Physics, University of Eastern Finland, Kuopio 70211, Finland
[5] Department of orthopedics, traumatology, hand surgery, Kuopio University Hospital, Kuopio 70210, Finland
[6] Institute of Analytical and Bioanalytical Chemistry, Ulm University, Ulm 89081, Germany

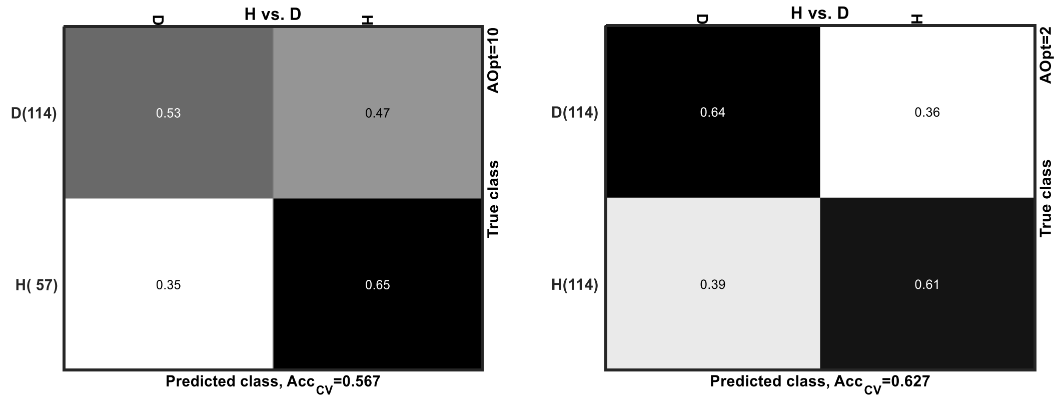

Figure S1: Classification results for the bovine data when no oversampling was applied (a) vs oversampled data (b). The samples of healthy group were simply duplicated. The results are presented for the sparse data of EMSC1 preprocessed broadband spectra. Similar results were observed for other sparse data. Acc_{CV} stands for Accuracy of cross-validation, A_{Opt} is the number of latent variables in the PLSDA model, H – healthy, D – damaged.

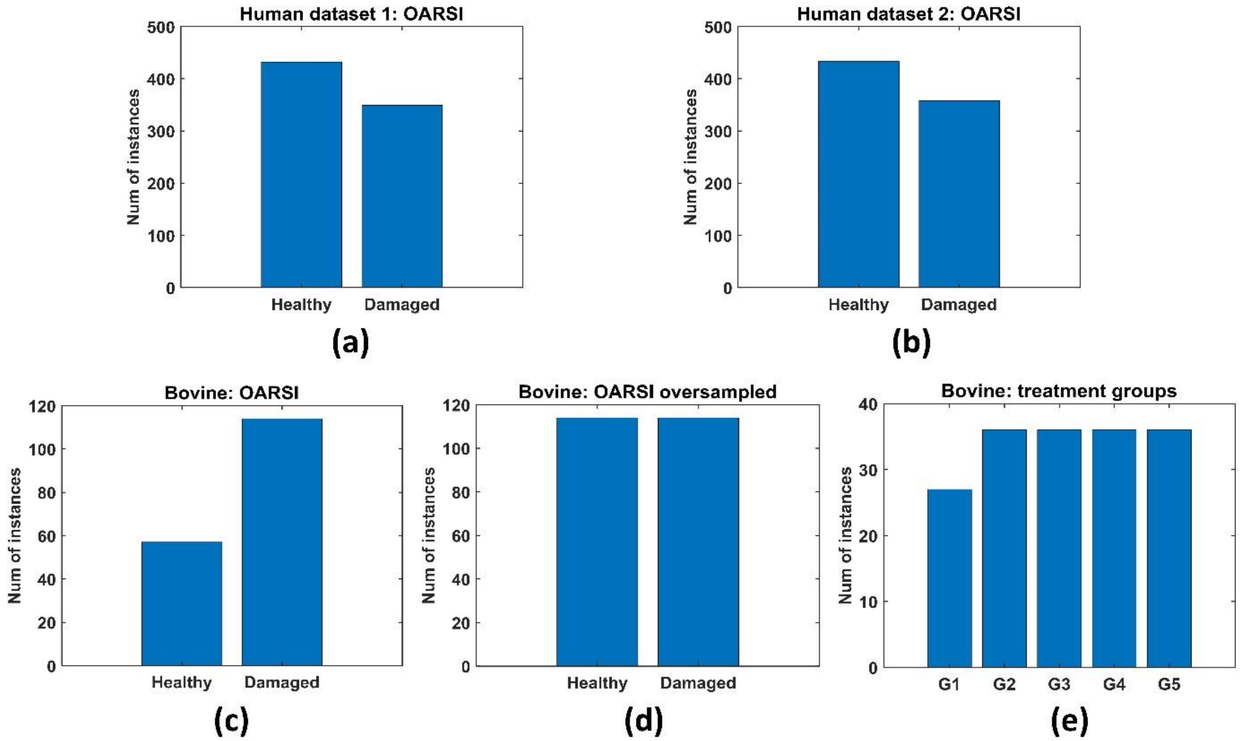

Figure S2: Distribution of samples in OARSI groups: healthy vs damaged of human 1 (a), human 2 (b) and bovine data before oversampling (c) and after oversampling (d). OARSI<= θ_{OARSI} belongs to healthy cartilage group, while grade OARSI> θ_{OARSI} is damaged group where $\theta_{OARSI} = 2$ for all datasets. Distribution of the samples in the treatment group for the bovine data in (e). The distributions of samples are provided for the quality tested datasets.

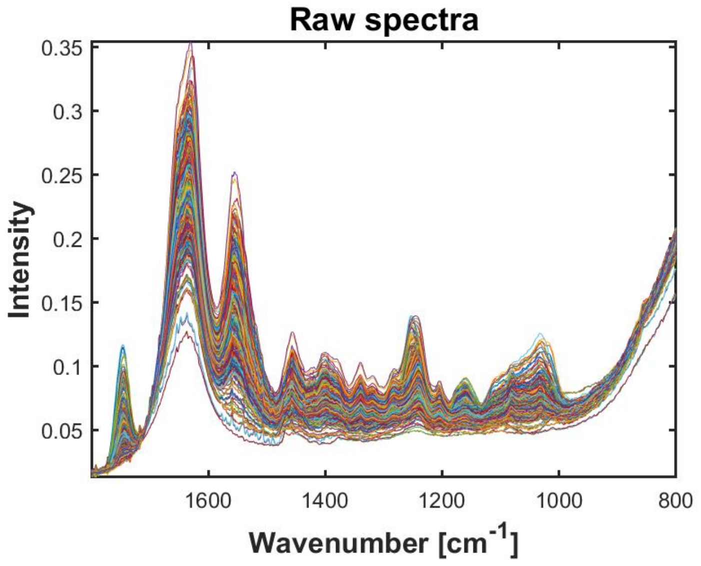

Figure S3: Raw broadband spectra of human dataset 1 samples after removing water spectra by spectral pre-classification method based on EMSC.

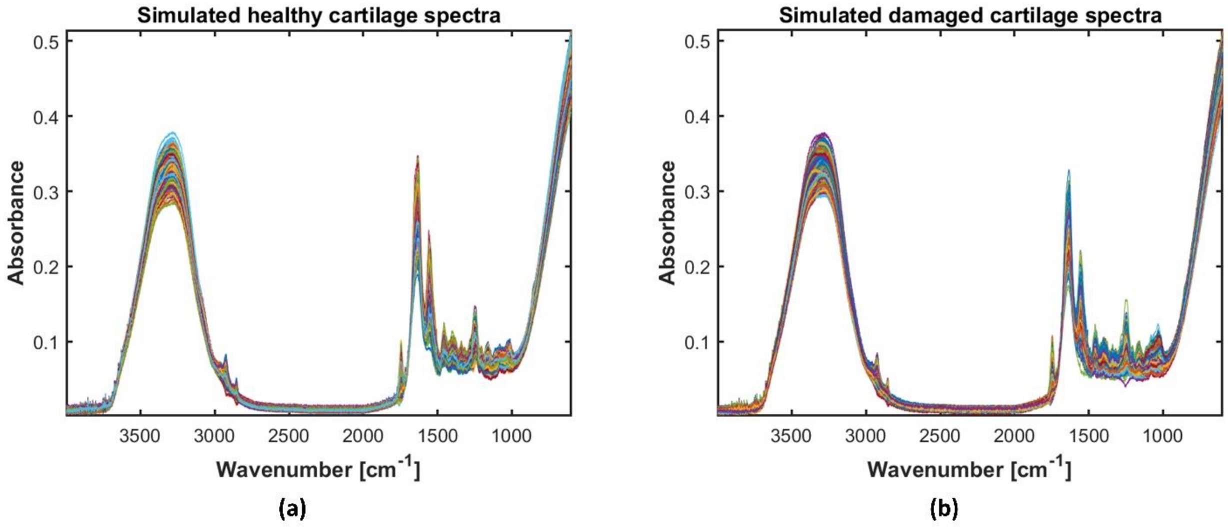

Figure S4: Simulated apparent spectra of human (a) healthy and (b) damaged cartilage.

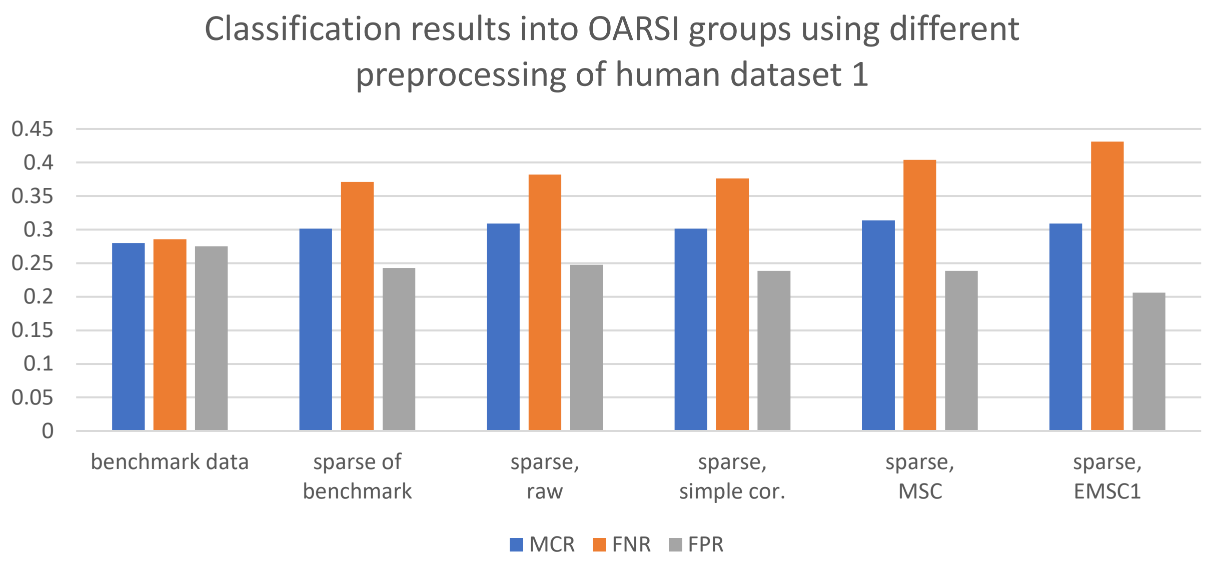

Figure S5: Binary PLSDA classification of healthy and damaged samples based on OARSI grades. Models are established using preprocessed spectra of human dataset 1. From left to right: (1) benchmark broadband data, (2) sparse spectra of the benchmark data, (3) sparse raw data, (3) sparse data with simple preprocessing, (4) sparse data preprocessed by MSC, (5) sparse data preprocessed by EMSC1. Overall misclassification rate (MCR = 1 - Accuracy) as well as False Negative Rate (FNR = 1 - Sensitivity) and False Positive Rate (FPR = 1 - Specificity) for the damaged group are provided.

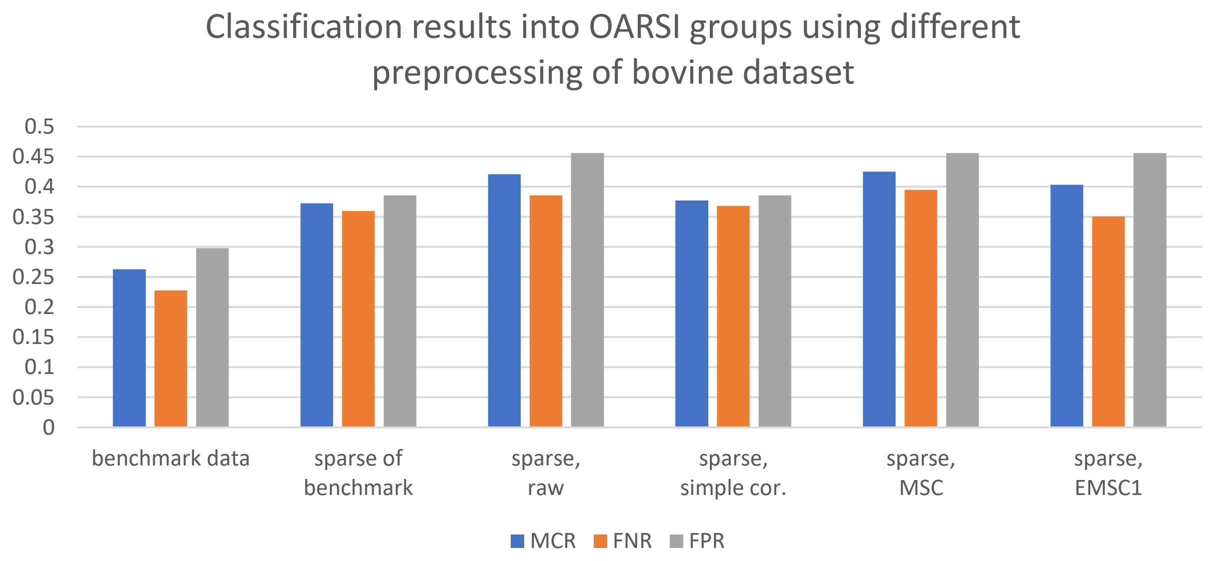

Figure S6: Binary PLSDA classification of healthy and damaged samples based on OARSI grades. Models are established using preprocessed spectra of bovine dataset. From left to right: (1) benchmark broadband data, (2) sparse spectra of the benchmark data, (3) sparse raw data, (3) sparse data with simple preprocessing, (4) sparse data preprocessed by MSC, (5) sparse data preprocessed by EMSC1. Overall misclassification rate (MCR = 1 - Accuracy) as well as False Negative Rate (FNR = 1 - Sensitivity) and False Positive Rate (FPR = 1 - Specificity) for the damaged group are provided.

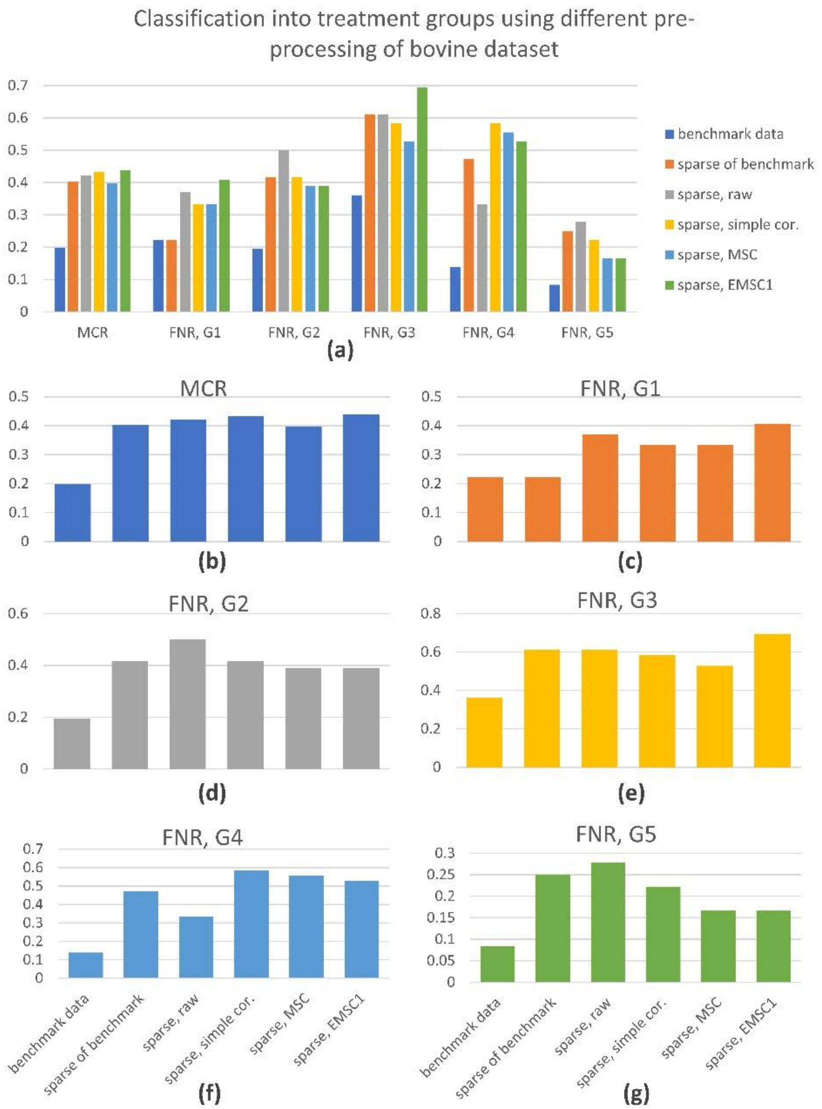

Figure S7: Multiclass classification into five treatment groups by PLSDA models established using preprocessed spectra of bovine samples. (a) All results combined for all groups G1-G5; (b)-(g) FNR for classes G1-G5, respectively. Different bars represent different preprocessing, from left to right: (1) benchmark broadband data, (2) sparse spectra of the benchmark data, (3) sparse data preprocessed by baseline offset at 1800 cm^{-1} and peak normalization at 850 cm^{-1}, (4) sparse data preprocessed by MSC, (5) sparse data preprocessed by EMSC1. Treatment groups correspond to three enzymatic damage groups: G1 – collagenase 24h treatment, G2 – collagenase 90 min treatment, G3 – trypsin30 min treatment, and two mechanical damage groups: G4 – surface abrasion, G5 – impact loading. Overall misclassification rate (MCR = 1 - Accuracy) as well as False Negative Rate (FNR = 1 - Sensitivity) and False Positive Rate (FPR = 1 - Specificity) for each class is presented.

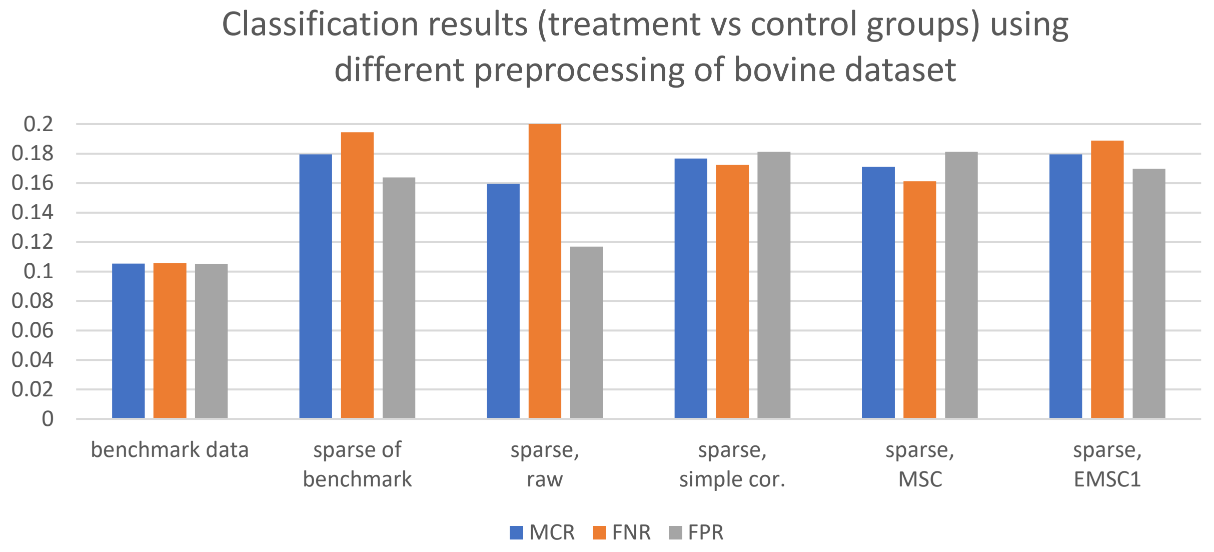

Figure S8: Results of binary classification into treatment and control groups by PLSDA models established using preprocessed spectra of bovine samples. From left to right: (1) benchmark broadband data, (2) sparse spectra of the benchmark data, (3) sparse data preprocessed by baseline offset at 1800 cm^{-1} and peak normalization at 850 cm^{-1}, (4) sparse data preprocessed by MSC, (5) sparse data preprocessed by EMSC1. Treatment groups correspond to three enzymatic damage groups: G1 – collagenase 24h treatment, G2 – collagenase 90 min treatment, G3 – trypsin30 min treatment, and two mechanical damage groups: G4 – surface abrasion, G5 – impact loading. Controls are the samples before the treatment has been done. Overall misclassification rate (MCR = 1- Accuracy) as well as False Negative Rate (FNR = 1 - Sensitivity) and False Positive Rate (FPR = 1- Specificity) for the control group are provided.

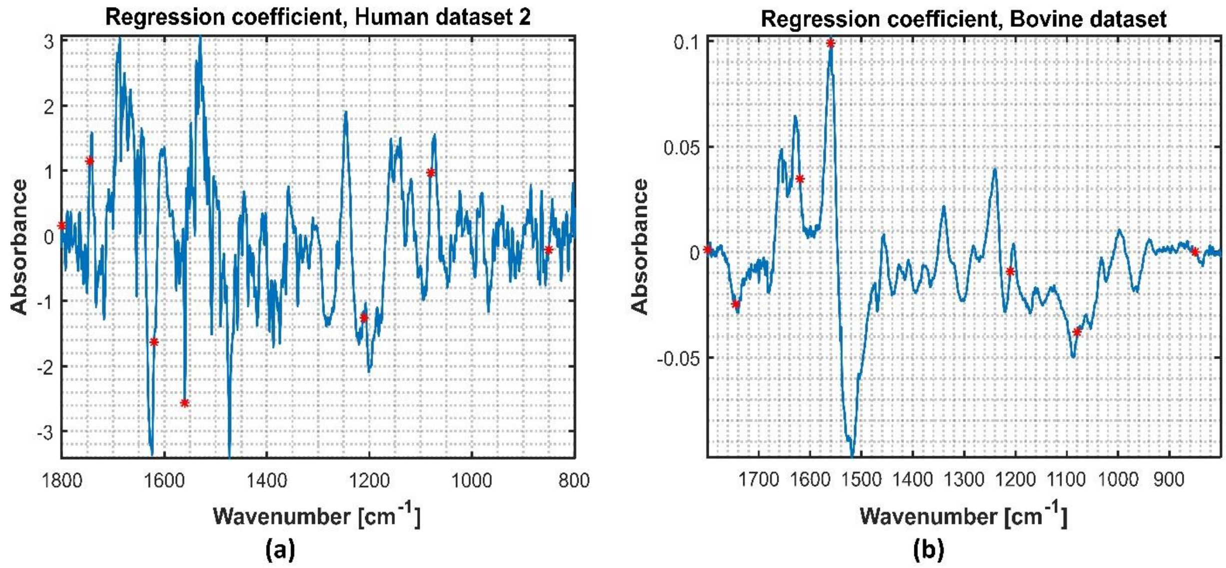

Figure S9: Regression coefficients of the PLSDA models obtained on the benchmark broadband spectra of human dataset 2 (a) and bovine dataset (b). Seven wavelengths of the sparse data are provided in red stars for visualization purposes.

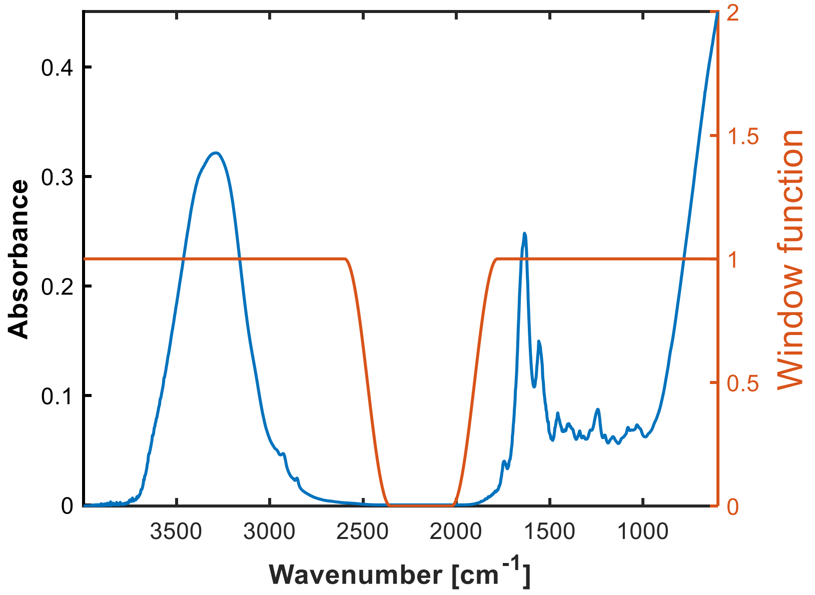

Figure S10: Window function Tukey used in simulating spectra.

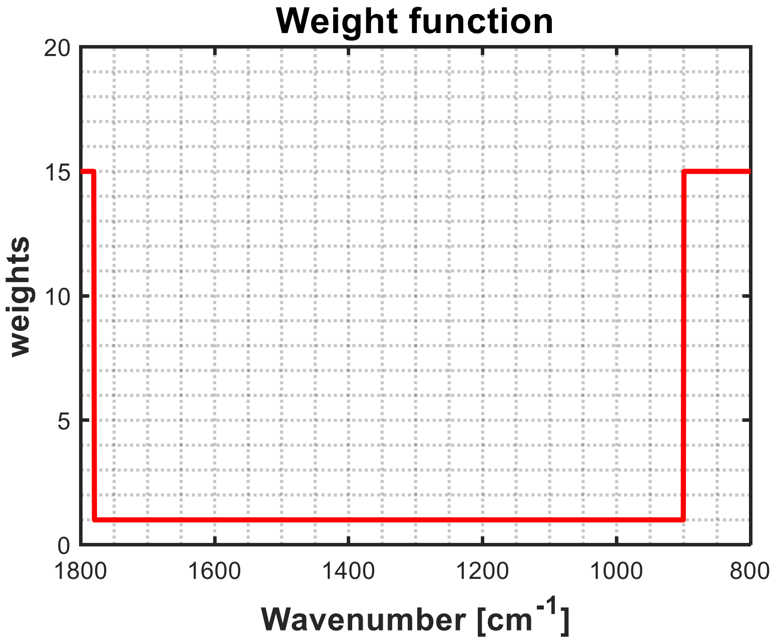

Figure S11: Wavenumber-depended weights used for preprocessing of broadband spectra by EMSC1 – MSC extended by linear term. Broadband spectra preprocessed by weighted EMSC1 is used as the benchmark data in this study.
